# Anticoagulant therapy and home blood pressure-associated risk for stroke/bleeding events in elderly patients with non-valvular atrial fibrillation: the sub-cohort study of ANAFIE registry

**DOI:** 10.1038/s41440-023-01361-4

**Published:** 2023-07-11

**Authors:** Kazuomi Kario, Naoyuki Hasebe, Ken Okumura, Takeshi Yamashita, Masaharu Akao, Hirotsugu Atarashi, Takanori Ikeda, Yukihiro Koretsune, Wataru Shimizu, Shinya Suzuki, Hiroyuki Tsutsui, Kazunori Toyoda, Atsushi Hirayama, Masahiro Yasaka, Takenori Yamaguchi, Satoshi Teramukai, Tetsuya Kimura, Yoshiyuki Morishima, Atsushi Takita, Hiroshi Inoue

**Affiliations:** 1 Jichi Medical University, Tochigi, Japan; 2 Asahikawa Medical University, Hokkaido, Japan; 3 Saiseikai Kumamoto Hospital Cardiovascular Center, Kumamoto, Japan; 4 The Cardiovascular Research Institute, Tokyo, Japan; 5 National Hospital Organization Kyoto Medical Center, Kyoto, Japan; 6 AOI Hachioji Hospital, Tokyo, Japan; 7 Toho University Faculty of Medicine, Tokyo, Japan; 8 National Hospital Organization Osaka National Hospital, Osaka, Japan; 9 Nippon Medical School, Tokyo, Japan; 10 Kyushu University, Fukuoka, Japan; 11 National Cerebral and Cardiovascular Center, Osaka, Japan; 12 Osaka Police Hospital, Osaka, Japan; 13 National Hospital Organization Kyushu Medical Center, Fukuoka, Japan; 14 Kyoto Prefectural University of Medicine, Kyoto, Japan; 15 Daiichi Sankyo Co., Ltd., Tokyo, Japan; 16 Saiseikai Toyama Hospital, Toyama, Japan

**Keywords:** Anticoagulants, Atrial fibrillation, Home blood pressure, Elderly

## Abstract

The benefits of direct oral anticoagulants (DOACs) and warfarin in elderly Japanese patients with non-valvular atrial fibrillation (NVAF) and high home systolic blood pressure (H-SBP) are unclear. This sub-cohort study of the ANAFIE Registry estimated the incidence of clinical outcomes in patients receiving anticoagulant therapy (warfarin and DOACs) stratified by H-SBP levels (<125 mmHg, ≥125–<135 mmHg, ≥135–<145 mmHg and ≥145 mmHg). Of the overall ANAFIE population, 4933 patients who underwent home blood pressure (H-BP) measurements were analyzed; 93% received OACs (DOACs: 3494, 70.8%; warfarin: 1092, 22.1%). In the warfarin group, at <125 mmHg and ≥145 mmHg, the respective incidence rates (per 100 person-years) were 1.91 and 5.89 for net cardiovascular outcome (a composite of stroke/systemic embolic events (SEE) and major bleeding), 1.31 and 3.39 for stroke/SEE, 0.59 and 3.91 for major bleeding, 0.59 and 3.43 for intracranial hemorrhage (ICH), and 4.01 and 6.24 for all-cause death. Corresponding incidence rates in the DOACs group were 1.64 and 2.65, 1.00 and 1.88, 0.78 and 1.69, 0.55 and 1.31, and 3.43 and 3.51. In warfarin-treated patients, the incidence rates of net cardiovascular outcome, stroke/SEE, major bleeding, and ICH were significantly increased at H-SBP ≥ 145 mmHg versus <125 mmHg. In the DOAC group, although there was no significant difference between H-SBP < 125 mmHg and ≥145 mmHg, the incidence rates of these events tended to increase at ≥145 mmHg. These results suggest that strict BP control guided by H-BP is required in elderly NVAF patients receiving anticoagulant therapy.

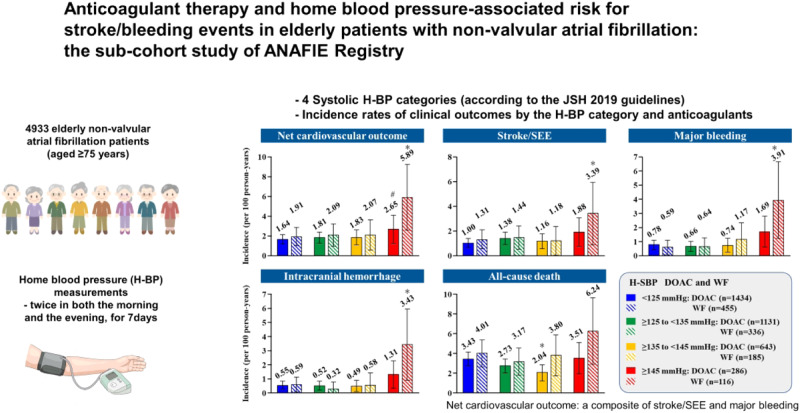

## Introduction

Atrial fibrillation (AF) is a major risk factor for ischemic stroke, affecting the life expectancy of elderly patients [1]. In patients with AF, 56.5% of patients reportedly have >1 modifiable risk factor, of which the most relevant is hypertension [2]. Furthermore, in patients with AF, hypertension is a risk factor for both embolism and bleeding complications and is a modifiable component of CHADS_2_ and HAS-BLED scores [2].

The All Nippon AF in the Elderly (ANAFIE) Registry was a 2-year multicenter, prospective observational study conducted to clarify the prognosis and real-world clinical status of over 30,000 elderly patients (aged ≥75 years) with non-valvular AF (NVAF) [3]. A sub-cohort study of the ANAFIE Registry reported that high home blood pressure (H-BP) was associated with an increased risk of stroke/systemic embolic events (SEE), major bleeding, and intracranial hemorrhage (ICH) [4].

Recently, direct oral anticoagulants (DOACs) have been used more frequently for stroke prevention in patients with NVAF in clinical practice than warfarin. Sub-analyses of multiple phase III clinical studies of DOACs have shown that the relative efficacy and safety of DOACs versus warfarin were consistent across all levels of systolic BP (SBP) [5–8]. The relationship between BP and the efficacy and safety of DOACs versus warfarin is not fully known in elderly NVAF patients in real-world clinical practice. In particular, the benefits of DOACs compared with warfarin in patients with high H-BP who are at increased risk of events are unclear.

A previous report of an H-BP sub-cohort study [4] showed the impact of high H-BP on the risk of clinical outcomes but did not cover the relationship between anticoagulant use and the risk of clinical outcomes. This analysis of the H-BP sub-cohort study of the ANAFIE Registry aimed to clarify the incidence of each clinical outcome in elderly NVAF patients receiving oral anticoagulant therapy (i.e., warfarin or DOACs) stratified by H-BP level.

Point of View

- Clinical relevance
  For Asian patients, particularly elderly Japanese patients with non-valvular atrial fibrillation (NVAF) undergoing therapy with oral anticoagulants (OACs), strict blood pressure control, guided by home blood pressure measurements (H-BP), may help decrease the risk of negative events such as net cardiovascular outcome, stroke/systemic embolic events, major bleeding, and intracranial hemorrhage.
- Future direction
  Future prospective studies evaluating the effects of direct OACs versus warfarin for elderly Asian patients with NVAF according to H-BP measurements considering changes in OACs and H-BP during the observation period are warranted.
- Consideration for the Asian population
  Although this sub-cohort study of the ANAFIE Registry included only Japanese patients, we consider that these results may apply to other Asian populations and may provide the information for hypertension management in elderly Asian patients with NVAF.

## Methods

### Design

This is a sub-cohort study of the ANAFIE Registry, for which the methods and rationale have been described previously [4, 9]. Ethical approval was obtained from all relevant institutional review boards and procedures were in accordance with the Declaration of Helsinki. All participants provided written informed consent to participate and could withdraw at any time. The ANAFIE Registry was registered at UMIN Clinical Trials Registry under the identifier UMIN000024006.

Of note, the choice to prescribe oral anticoagulant (OAC) therapy or no OAC therapy, warfarin, or DOACs was at the discretion of the physician. Baseline data were used for analyses. Patients were followed up for 2 years from enrollment.

### Patients

Patients aged ≥75 years at the time of informed consent with a definitive NVAF diagnosis who could attend hospital visits were included. For enrollment in the current sub-cohort study, patients needed to consent to measure their H-BP using an oscillometric device with an arm cuff. Patients with a definite diagnosis of mitral stenosis; an artificial heart valve replacement; very recent history (within 1 month prior to enrollment) of cardiovascular events, including stroke, myocardial infarction, cardiac intervention, heart failure requiring hospitalization, or any bleeding leading to hospitalization; or life expectancy <1 year were excluded from enrollment in this study [4].

### Endpoints

The study endpoints were net cardiovascular outcome (a composite of stroke/SEE and major bleeding), stroke/SEE, major bleeding, ICH, and all-cause death. Any events during follow-up were recorded in duplicate. Committees comprising neurologists, cardiologists, and hematologists blinded to the anticoagulation treatment adjudicated all endpoint events. Major bleeding was classified using the International Society on Thrombosis and Haemostasis definition [4, 10].

### Data collection method

H-BP measurements were based on previously described procedures [4, 11]. Measurements were taken four times a day (twice in the morning and twice in the evening) for 1 week within 60 days of enrollment in line with Japanese Society of Hypertension (JSH) guidelines [12] and recorded. The average of all measurements over 1 week was used for analysis. Participants could use any device based on the brachial cuff oscillometric method.

### Statistical analysis

Details of the statistical analysis for the main ANAFIE Registry have been published [3, 4]. The following H-SBP categories based on JSH guidelines [12] were used: <125 mmHg, ≥125–<135 mmHg, ≥135–<145 mmHg, and ≥145 mmHg. Patient characteristics by H-SBP category were compared with analysis of variance for continuous variables and the chi-square test for categorical variables. However, unknown data were excluded from the analysis. The incidence rate per 100 person-years with 95% confidence intervals (CIs) was estimated. The incidence-rate ratio (relative risk) was estimated using the Poisson regression model. The hazard ratios (HRs) and 95% CIs of DOACs compared with warfarin for the clinical outcomes were analyzed with a multivariate Cox proportional hazards model for each H-SBP category. The following variables were included in the model as covariates: sex, age, body mass index, history of major bleeding, atrial fibrillation type, severe hepatic dysfunction, diabetes mellitus, hyperuricemia, heart disease (heart failure and/or left ventricular systolic dysfunction), myocardial infarction, cerebrovascular disease, thromboembolic-related disease, active cancer, dementia, falls within 1 year, nonpharmacologic therapy (catheter ablation), antiarrhythmics, antiplatelet agents, proton pump inhibitors, P-glycoprotein inhibitors, dyslipidemia, creatinine clearance, gastrointestinal disease, and polypharmacy. The H-SBP by treatment (DOACs versus warfarin) interaction effect was also estimated. The statistical analysis software used was SAS version 9.4 (SAS Institute, Tokyo, Japan), and the significance level was *p* < 0.05.

## Results

### Patient disposition and background characteristics

Of the overall ANAFIE population (*N* = 32,275), 1109 (3.4%) patients were lost to follow-up and 762 (2.4%) discontinued the study because of withdrawal of consent and other reasons [3]. In this H-BP sub-cohort, 4933 patients were included who provided written consent to be enrolled and measured their H-BP. The mean follow-up period was 1.88 years [3].

Table 1 shows patient characteristics at baseline. The patient breakdown by H-SBP category was as follows: 2030, 1585, 878, and 440 in the <125 mmHg, ≥125–<135 mmHg, ≥135–<145 mmHg and ≥145 mmHg groups, respectively. The mean age was 81.4 years, and over 50% were male as a whole. Most patients (4590/4933; 93%) were receiving anticoagulants. Of these, 3494 (70.8%) received DOACs, 1092 (22.1%) received warfarin, and there was no difference in the distribution of OACs between H-SBP groups. Across groups, the mean CHA_2_DS_2_-VASc and HAS-BLED scores significantly increased as H-SBP increased. The rate of antihypertensive drug use was significantly different between the H-SBP groups. We analyzed the differences in patient backgrounds between the warfarin and DOAC groups in each H-SBP category (Supplementary Table 1).

**Table 1:** Patient characteristics at baseline by H-SBP category

Characteristic	Overall *n* = 4933	<125 mmHg *n* = 2030	≥125–<135 mmHg *n* = 1585	≥135–<145 mmHg *n* = 878	≥145 mmHg *n* = 440	*p* value^a^
Age, years	81.4 ± 4.8	81.1 ± 4.9	81.3 ± 4.6	81.7 ± 4.9	82.2 ± 4.9	<0.001
Male	2770 (56.2)	1103 (54.3)	904 (57.0)	504 (57.4)	259 (58.9)	0.165
Body mass index, kg/m^2^	23.4 ± 3.6	23.1 ± 3.6	23.4 ± 3.6	23.9 ± 3.6	23.8 ± 3.5	<0.001
Anticoagulants	4590 (93.0)	1891 (93.2)	1467 (92.6)	829 (94.4)	403 (91.6)	0.201
Warfarin	1092 (22.1)	455 (22.4)	336 (21.2)	185 (21.1)	116 (26.4)	0.064
DOAC	3494 (70.8)	1434 (70.6)	1131 (71.4)	643 (73.2)	286 (65.0)	0.053
Dabigatran	357 (7.2)	156 (7.7)	109 (6.9)	64 (7.3)	28 (6.4)	0.747
Rivaroxaban	1184 (24.0)	464 (22.9)	397 (25.0)	226 (25.7)	97 (22.0)	0.226
Apixaban	1184 (24.0)	509 (25.1)	385 (24.3)	194 (22.1)	96 (21.8)	0.195
Edoxaban	769 (15.6)	305 (15.0)	240 (15.1)	159 (18.1)	65 (14.8)	0.231
Antihypertensives	3658 (77.1)	1431 (73.2)	1186 (78.0)	685 (81.1)	356 (84.0)	<0.001
Antiplatelet drugs	827 (17.4)	336 (17.2)	265 (17.4)	142 (16.8)	84 (19.8)	0.575
CHA_2_DS_2_-VASc score	4.4 ± 1.3	4.4 ± 1.4	4.4 ± 1.3	4.4 ± 1.3	4.6 ± 1.3	0.014
HAS-BLED score	1.8 ± 0.8	1.8 ± 0.8	1.8 ± 0.8	1.9 ± 0.9	1.9 ± 0.9	0.002
Creatinine clearance, mL/min	49.1 ± 17.1	47.9 ± 17.1	50.6 ± 16.7	50.3 ± 17.8	47.7 ± 17.0	<0.001
<50 mL/min	2180 (44.2)	944 (46.5)	658 (41.5)	366 (41.7)	212 (48.2)	<0.001
Atrial fibrillation type						
Paroxysmal	2052 (41.6)	783 (38.6)	697 (44.0)	382 (43.5)	190 (43.2)	<0.001
Non-paroxysmal	2881 (58.4)	1247 (61.4)	888 (56.0)	496 (56.5)	250 (56.8)	
Comorbidities	4800 (97.3)	1975 (97.3)	1540 (97.2)	853 (97.2)	432 (98.2)	0.684
History of major bleeding	163 (3.3)	68 (3.3)	53 (3.3)	31 (3.5)	11 (2.5)	0.789
Cerebrovascular diseases	1112 (22.5)	449 (22.1)	342 (21.6)	213 (24.3)	108 (24.5)	0.315
Dyslipidemia	2132 (43.2)	869 (42.8)	713 (45.0)	370 (42.1)	180 (40.9)	0.318
Diabetes mellitus	1278 (25.9)	469 (23.1)	423 (26.7)	251 (28.6)	135 (30.7)	<0.001
Chronic kidney disease	926 (18.8)	415 (20.4)	265 (16.7)	170 (19.4)	76 (17.3)	0.030
Cardiac diseases	2725 (55.2)	1212 (59.7)	805 (50.8)	452 (51.5)	256 (58.2)	<0.001

Data are *n* (%) or mean ± standard deviation

*DOAC* direct oral anticoagulant, *H-SBP* home systolic blood pressure

^a^Comparison among four blood pressure groups

### Outcomes

**Fig. 1:**
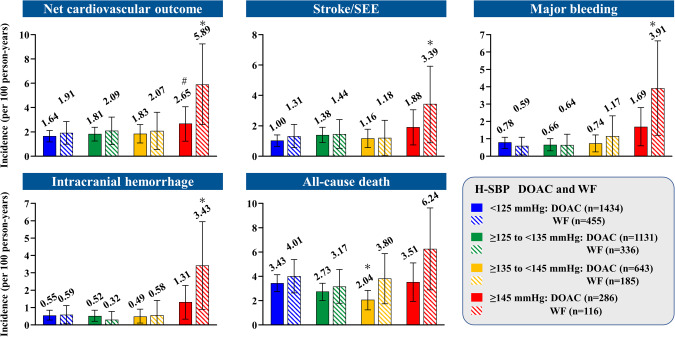
Incidence rates (per 100 person-years) of net cardiovascular outcome, stroke/SEE, major bleeding, intracranial hemorrhage, and all-cause death during follow-up in the DOAC and warfarin groups according to H-SBP. Bars represent 95% confidence intervals. *p < 0.05 versus H-SBP < 125 mmHg in each OAC group, #*p* < 0.05 versus warfarin. DOAC indicates direct oral anticoagulant, H-SBP home systolic blood pressure, SEE systemic embolic events, WF warfarin

Overall, during the 2 years of follow-up, there were 172 cases of net cardiovascular outcomes; 115, stroke/SEE; 76, major bleeding; 57, ICH; and 299, all-cause death. Supplementary Table 2 shows the number of events of the warfarin group and the DOAC group. Figure 1 shows the incidence rates (per 100 person-years) of net cardiovascular outcome, stroke/SEE, major bleeding, ICH, and all-cause death during follow-up in the warfarin and DOAC groups, according to H-SBP. In the warfarin group, the respective incidence rates among patients with H-SBP < 125 mmHg and ≥145 mmHg were 1.91 and 5.89 for net cardiovascular outcome, 1.31 and 3.39 for stroke/SEE, 0.59 and 3.91 for major bleeding, 0.59 and 3.43 for ICH, and 4.01 and 6.24 for all-cause death (Fig. 1). The incidence rates of net cardiovascular outcome, stroke/SEE, major bleeding, and ICH were significantly increased at H-SBP ≥ 145 mmHg versus <125 mmHg (*p* < 0.05).

In the DOAC group, among patients with <125 mmHg and ≥145 mmHg, the respective incidence rates were 1.64 and 2.65 for net cardiovascular outcome, 1.00 and 1.88 for stroke/SEE, 0.78 and 1.69 for major bleeding, 0.55 and 1.31 for ICH and 3.43 and 3.51 for all-cause death. Although the incidence rate of these events tended to increase with H-SBP ≥ 145 mmHg, there was no significant difference in the event rates between H-SBP < 125 mmHg and ≥145 mmHg in the DOAC group. In comparison with the warfarin group, the incidence rate of net cardiovascular outcome was significantly lower in the DOAC group at H-SBP ≥ 145 mmHg (*p* < 0.05). In patients with H-SBP ≥ 145 mmHg, CHA_2_DS_2_-VASc score in the warfarin group was significantly higher than that in the DOAC group, whereas there was no significant difference in HAS-BLED score between the groups (Supplementary Table 1).

Supplementary Table 3 shows the incidence rates of net cardiovascular outcome, stroke/SEE, major bleeding, ICH, and all-cause death during follow-up in the DOAC group according to H-SBP, excluding patients who received off-label doses of DOACs. There was no significant difference in the incidence rates of outcomes at any SBP level except for ICH (*p* = 0.045) at H-SBP ≥ 145 mmHg.

**Fig. 2:**
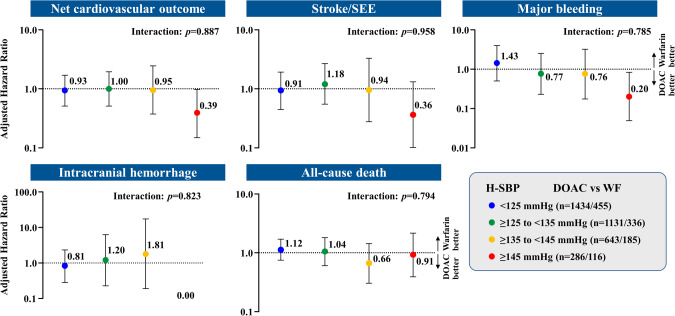
Forest plots of adjusted hazard ratios for patients treated with DOACs versus warfarin for net cardiovascular outcome, stroke/SEE, major bleeding, intracranial hemorrhage, and all-cause death. Bars represent 95% confidence intervals. Abbreviations are the same as in Fig. 1

Figure 2 shows the adjusted HRs for patients treated with DOACs versus warfarin for net cardiovascular outcome, stroke/SEE, major bleeding, ICH, and all-cause death. There was no significant interaction between H-SBP and the relative effectiveness and safety of DOACs versus warfarin.

Supplementary Table 4 shows adjusted HRs of DOAC versus warfarin in the four H-SBP categories, excluding patients who received off-label doses of DOACs, and the result was similar to that of Fig. 2.

## Discussion

In this sub-analysis of a prespecified sub-cohort study of the ANAFIE Registry [4], we found that the incidence rates of net cardiovascular outcome, stroke/SEE, major bleeding, and ICH were significantly higher among patients with H-SBP ≥ 145 mmHg than those with H-SBP < 125 mmHg in the warfarin group. In the DOAC group, the incidence rates of these events also increased numerically, although the difference was not statistically significant. These results suggest that in elderly NVAF patients, strict BP control guided by H-BP is required, irrespective of the type of anticoagulation used. Meanwhile, the incidences of stroke/SEE, major bleeding, and ICH tended to be lower with DOACs than with warfarin among patients with H-SBP ≥ 145 mmHg, suggesting the merit of DOAC use for elderly AF patients, especially when the strict control of H-SBP is difficult.

The BAT study [13] (comprising patients both with and without AF), Fushimi AF Registry [14], and J-RHYTHM Registry [15] were all conducted during a time when warfarin was the predominant anticoagulant prescribed for stroke prevention, suggesting that the association of hypertension with major bleeding and ICH observed in these studies was perhaps at least partly a consequence of warfarin use.

The ENGAGE AF-TIMI 48 trial reported similar efficacy between edoxaban and warfarin in patients with AF and a history of hypertension, regardless of SBP stratification [5]. Edoxaban significantly reduced the incidence of major bleeding events and ICH versus warfarin across all SBP groups. The relative safety of edoxaban was most pronounced in patients with elevated diastolic BP. In a sub-analysis of the ROCKET AF trial, the benefit of rivaroxaban and warfarin to prevent stroke or systemic embolism was similar and did not vary by SBP group [8]. Similarly, the ARISTOTLE trial reported similar benefits for apixaban and warfarin for the prevention of stroke/SEE across BP groups [7]. In the RE-LY trial, there was no significant difference in the efficacy of dabigatran versus warfarin in hypertensive patients with NVAF for the incidence of stroke/SEE or major bleeds [5].

This study has some limitations that must be considered when interpreting the findings. The limitations of the observational ANAFIE Registry and this sub-cohort study have been described previously [3, 4]. Specifically, data on DOAC and warfarin use and H-BP were collected at baseline only, and any changes in OACs and antihypertensive drugs during the observation period were not considered.

### Perspectives in Asia

In Asian countries with a disproportionate growth of aging populations and increased risk of comorbidities and complications, the benefits of anticoagulation in elderly NVAF patients with high BP should be considered against the risk of clinical outcomes. A review by Yasaka and Lip reported that the incidence of ICH is markedly higher in Japan and other East Asian countries than in countries outside East Asia [16]. In the current study, the incidence rates (per 100 person-years) of ICH at ≥145 mmHg were 3.43 and 1.31 in the warfarin and DOAC groups, respectively. The ICH incidence rates at H-SBP ≥ 145 mmHg appeared to be higher in this sub-cohort study than in global studies such as the ENGAGE AF-TIMI 48 trial [6]. Thus, strict BP control may be particularly important for elderly Asian patients with NVAF undergoing anticoagulant therapy.

## Conclusions

Among elderly NVAF patients treated with warfarin, the incidence rates of net cardiovascular outcome, stroke/SEE, major bleeding, and ICH were significantly increased at H-SBP ≥ 145 mmHg versus <125 mmHg, but not in those treated with DOACs. Among elderly patients ≥75 years of age with NVAF who are receiving either anticoagulation with DOACs or warfarin, strict BP control guided by H-BP may be required.

### Supplementary information

Supplementary Tables
