# Supplementary material for: Anticoagulant therapy and home blood pressure-associated risk for stroke/bleeding events in elderly patients with non-valvular atrial fibrillation: the sub-cohort study of ANAFIE registry
Source: Hypertens Res. 2023 Jul 11;46(12):2575–82. doi: 10.1038/s41440-023-01361-4 (PMC10695825; doi:10.1038/s41440-023-01361-4)
Supplement: Supplementary file 1 — Supplementary Tables [file 41440_2023_1361_MOESM1_ESM.docx]

**Tables**

**Supplementary Table 1.** Patient characteristics at baseline in the warfarin and DOAC groups by H-SBP category

| Characteristic | Overall | | | <125 mmHg | | | ≥125 to <135 mmHg | | | ≥135 to <145 mmHg | | | ≥145 mmHg | | |
| --- | --- | --- | --- | --- | --- | --- | --- | --- | --- | --- | --- | --- | --- | --- | --- |
|  | Warfarin  n=1092 | DOAC  n=3494 | *p*-value^a^ | Warfarin  n=455 | DOAC  n=1434 | *p*-value^a^ | Warfarin  n=336 | DOAC  n=1131 | *p*-value^a^ | Warfarin  n=185 | DOAC  n=643 | *p*-value^a^ | Warfarin  n=116 | DOAC  n=286 | *p*-value^a^ |
| Age, years | 81.6±5.0 | 81.2±4.6 | 0.005 | 81.1±4.9 | 81.0±4.7 | 0.707 | 81.6±4.7 | 81.1±4.5 | 0.059 | 82.8±5.2 | 81.4±4.7 | 0.001 | 82.2±5.2 | 82.0±4.7 | 0.682 |
| Male | 650  (59.5) | 1,959  (56.1) | 0.044 | 254  (55.8) | 784  (54.7) | 0.667 | 204  (60.7) | 647  (57.2) | 0.253 | 119  (64.3) | 361  (56.1) | 0.0470 | 73  (62.9) | 167  (58.4) | 0.4005 |
| Body mass index, kg/m^2^ | 23.2  ±3.6 | 23.5  ±3.6 | 0.021 | 22.8  ±3.8 | 23.2  ±3.5 | 0.024 | 23.3  ±3.3 | 23.6  ±3.6 | 0.1678 | 23.3  ±3.5 | 24.0  ±3.6 | 0.054 | 24.6  ±3.9 | 23.5  ±3.4 | 0.016 |
| Antihypertensives | 828  (78.4) | 2,576  (76.7) | 0.240 | 317  (72.5) | 1,015  (73.3) | 0.743 | 260  (79.8) | 837  (77.3) | 0.347 | 151  (84.4) | 497  (80.3) | 0.220 | 100  (87.7) | 227  (82.8) | 0.230 |
| Antiplatelet drugs | 221  (20.9) | 475  (14.1) | <0.001 | 85  (19.5) | 200  (14.5) | 0.012 | 70  (21.5) | 151  (13.9) | 0.001 | 40  (22.3) | 85  (13.7) | 0.005 | 26  (22.8) | 39  (14.2) | 0.039 |
| CHA_2_DS_2_-VASc score | 4.6±1.4 | 4.4±1.3 | <0.001 | 4.5±1.4 | 4.4±1.4 | 0.037 | 4.5±1.4 | 4.3±1.3 | 0.003 | 4.5±1.4 | 4.4±1.3 | 0.554 | 4.8±1.3 | 4.5±1.4 | 0.036 |
| HAS-BLED score | 1.9±0.8 | 1.8±0.8 | <0.001 | 1.9±0.8 | 1.8±0.8 | <0.001 | 1.9±0.9 | 1.7±0.8 | 0.012 | 1.9±0.9 | 1.8±0.9 | 0.623 | 2.0±0.8 | 1.9±0.9 | 0.248 |
| Creatinine clearance, mL/min | 45.8±17.9 | 50.5±16.6 | <0.001 | 44.2±16.9 | 49.4±16.7 | <0.001 | 47.8±18.2 | 51.8±16.1 | <0.001 | 45.7±19.1 | 51.5±17.2 | <0.001 | 46.4±18.8 | 48.9±15.9 | 0.210 |
| <50 mL/min | 521  (47.8) | 1494  (42.8) | <0.001 | 233  (51.3) | 644  (44.8) | <0.001 | 149  (44.4) | 453  (40.1) | <0.001 | 85  (45.9) | 260  (40.4) | <0.001 | 54  (46.6) | 137  (47.8) | <0.001 |
| Atrial fibrillation type |  |  |  |  |  |  |  |  |  |  |  |  |  |  |  |
| Paroxysmal | 334  (30.6) | 1492  (42.7) | <0.001 | 138  (30.3) | 563  (39.3) | <0.001 | 102  (30.4) | 511  (45.2) | <0.001 | 63  (34.1) | 285  (44.3) | 0.001 | 31  (26.7) | 133  (46.5) | <0.001 |
| Non-paroxysmal | 758  (69.4) | 2002  (57.3) |  | 317  (69.7) | 871  (60.7) |  | 234  (69.7) | 620  (54.9) |  | 122  (66.0) | 358  (55.7) |  | 85  (73.2) | 153  (53.5) |  |
| Comorbidities | 1,061  (97.2) | 3,398  (97.3) | 0.873 | 443  (97.4) | 1,393  (97.1) | 0.803 | 324  (96.4) | 1,100  (97.3) | 0.428 | 180  (97.3) | 625  (97.2) | 0.944 | 114  (98.3) | 280  (97.9) | 0.808 |
| History of major bleeding | 36  (3.3) | 106  (3.0) | 0.662 | 17  (3.7) | 41  (2.9) | 0.345 | 13  (3.9) | 36  (3.2) | 0.539 | 6  (3.2) | 21  (3.3) | 0.988 | 0  (0.0) | 8  (2.8) | 0.069 |
| Cerebrovascular diseases | 237  (21.7) | 797  (22.8) | 0.445 | 100  (22.0) | 317  (22.1) | 0.954 | 72  (21.4) | 243  (21.5) | 0.982 | 41  (22.2) | 165  (25.7) | 0.332 | 24  (20.7) | 72  (25.2) | 0.339 |
| Dyslipidemia | 475  (43.5) | 1,492  (42.7) | 0.643 | 200  (44.0) | 605  (42.2) | 0.507 | 152  (45.2) | 502  (44.4) | 0.783 | 74  (40.0) | 271  (42.1) | 0.602 | 49  (42.2) | 114  (39.9) | 0.660 |
| Diabetes mellitus | 327  (29.9) | 871  (24.9) | 0.001 | 112  (24.6) | 330  (23.0) | 0.482 | 107  (31.8) | 287  (25.4) | 0.019 | 63  (34.1) | 178  (27.7) | 0.093 | 45  (38.8) | 76  (26.6) | 0.016 |
| Chronic kidney disease | 249  (22.8) | 624  (17.9) | <0.001 | 115  (25.3) | 281  (19.6) | 0.010 | 74  (22.0) | 172  (15.2) | 0.003 | 36  (19.5) | 126  (19.6) | 0.967 | 24  (20.7) | 45  (15.7) | 0.233 |
| Cardiac diseases | 701  (64.2) | 1,837  (52.6) | <0.001 | 306  (67.3) | 829  (57.8) | <0.001 | 203  (60.4) | 539  (47.7) | <0.001 | 116  (62.7) | 312  (48.5) | <0.001 | 76  (65.5) | 157  (54.9) | 0.051 |

Data are n (%) or mean ± standard deviation.

^a^Comparison among warfarin versus DOAC.

DOAC, direct oral anticoagulant; H-SBP, home systolic blood pressure.

**Supplementary Table 2.** The incidence of net CV outcome, stroke/SEE, major bleeding, ICH, and all-cause death during follow-up in the warfarin and DOAC groups by H-SBP category

|  |  | **Warfarin** | | | **DOAC** | | |
| --- | --- | --- | --- | --- | --- | --- | --- |
|  |  | N | Event number | Incidence proportion, % | N | Event number | Incidence proportion, % |
| Net CV outcome | Overall | 1092 | 48 | 4.40 | 3494 | 118 | 3.38 |
|  | H-SBP <125 mmHg | 455 | 16 | 3.52 | 1434 | 44 | 3.07 |
|  | H-SBP ≥125 to <135 mmHg | 336 | 13 | 3.87 | 1131 | 38 | 3.36 |
|  | H-SBP ≥135 to <145 mmHg | 185 | 7 | 3.78 | 643 | 22 | 3.42 |
|  | H-SBP ≥145 mmHg | 116 | 12 | 10.34 | 286 | 14 | 4.90 |
| Stroke/SEE | Overall | 1092 | 31 | 2.84 | 3494 | 80 | 2.29 |
|  | H-SBP <125 mmHg | 455 | 11 | 2.42 | 1434 | 27 | 1.88 |
|  | H-SBP ≥125 to <135 mmHg | 336 | 9 | 2.68 | 1131 | 29 | 2.56 |
|  | H-SBP ≥135 to <145 mmHg | 185 | 4 | 2.16 | 643 | 14 | 2.18 |
|  | H-SBP ≥145 mmHg | 116 | 7 | 6.03 | 286 | 10 | 3.50 |
| Major bleeding | Overall | 1092 | 21 | 1.92 | 3494 | 53 | 1.52 |
|  | H-SBP <125 mmHg | 455 | 5 | 1.10 | 1434 | 21 | 1.46 |
|  | H-SBP ≥125 to <135 mmHg | 336 | 4 | 1.19 | 1131 | 14 | 1.24 |
|  | H-SBP ≥135 to <145 mmHg | 185 | 4 | 2.16 | 643 | 9 | 1.40 |
|  | H-SBP ≥145 mmHg | 116 | 8 | 6.90 | 286 | 9 | 3.15 |
| ICH | Overall | 1092 | 16 | 1.47 | 3494 | 39 | 1.12 |
|  | H-SBP <125 mmHg | 455 | 5 | 1.10 | 1434 | 15 | 1.05 |
|  | H-SBP ≥125 to <135 mmHg | 336 | 2 | 0.60 | 1131 | 11 | 0.97 |
|  | H-SBP ≥135 to <145 mmHg | 185 | 2 | 1.08 | 643 | 6 | 0.93 |
|  | H-SBP ≥145 mmHg | 116 | 7 | 6.03 | 286 | 7 | 2.45 |
| All-cause death | Overall | 1092 | 80 | 7.33 | 3494 | 195 | 5.58 |
|  | H-SBP <125 mmHg | 455 | 34 | 7.47 | 1434 | 93 | 6.49 |
|  | H-SBP ≥125 to <135 mmHg | 336 | 20 | 5.95 | 1131 | 58 | 5.13 |
|  | H-SBP ≥135 to <145 mmHg | 185 | 13 | 7.03 | 643 | 25 | 3.89 |
|  | H-SBP ≥145 mmHg | 116 | 13 | 11.21 | 286 | 19 | 6.64 |

CI, confidence interval; CV, cardiovascular; DOAC, direct oral anticoagulant; H-SBP, home systolic blood pressure; ICH, intracranial hemorrhage; Ref, reference; SEE, systemic embolic events

**Supplementary Table 3**. The incidence rates of net CV outcome, stroke/SEE, major bleeding, ICH, and all-cause death during follow-up in the DOAC group by H-SBP category (analysis excludes patients with off-label DOAC doses)

|  |  | **DOAC (excluding off-label doses)** | | |
| --- | --- | --- | --- | --- |
|  |  | N | Incidence per 100 person-years (95% CI) | *p*-value^a^ |
| Net CV outcome | Overall | 2830 | 1.87 (1.50–2.24) |  |
|  | H-SBP <125 mmHg | 1163 | 1.60 (1.07–2.13) | Ref |
|  | H-SBP ≥125 to <135 mmHg | 926 | 1.97 (1.31–2.64) | 0.385 |
|  | H-SBP ≥135 to <145 mmHg | 509 | 2.00 (1.10–2.89) | 0.438 |
|  | H-SBP ≥145 mmHg | 232 | 2.56 (1.05–4.07) | 0.175 |
| Stroke/SEE | Overall | 2830 | 1.30 (0.99–1.61) |  |
|  | H-SBP <125 mmHg | 1163 | 1.05 (0.62–1.48) | Ref |
|  | H-SBP ≥125 to <135 mmHg | 926 | 1.44 (0.88–2.01) | 0.271 |
|  | H-SBP ≥135 to <145 mmHg | 509 | 1.25 (0.54–1.96) | 0.620 |
|  | H-SBP ≥145 mmHg | 232 | 2.09 (0.72–3.46) | 0.080 |
| Major bleeding | Overall | 2830 | 0.83 (0.58–1.07) |  |
|  | H-SBP <125 mmHg | 1163 | 0.68 (0.34–1.03) | Ref |
|  | H-SBP ≥125 to <135 mmHg | 926 | 0.81 (0.38–1.23) | 0.656 |
|  | H-SBP ≥135 to <145 mmHg | 509 | 0.83 (0.26–1.41) | 0.648 |
|  | H-SBP ≥145 mmHg | 232 | 1.61 (0.42–2.80) | 0.061 |
| ICH | Overall | 2830 | 0.64 (0.42–0.85) |  |
|  | H-SBP <125 mmHg | 1163 | 0.50 (0.20–0.80) | Ref |
|  | H-SBP ≥125 to <135 mmHg | 926 | 0.63 (0.26–1.00) | 0.586 |
|  | H-SBP ≥135 to <145 mmHg | 509 | 0.62 (0.12–1.12) | 0.663 |
|  | H-SBP ≥145 mmHg | 232 | 1.38 (0.28–2.49) | 0.045 |
| All-cause death | Overall | 2830 | 2.84 (2.38–3.29) |  |
|  | H-SBP <125 mmHg | 1163 | 3.08 (2.35–3.82) | Ref |
|  | H-SBP ≥125 to <135 mmHg | 926 | 2.63 (1.87–3.39) | 0.405 |
|  | H-SBP ≥135 to <145 mmHg | 509 | 2.27 (1.32–3.22) | 0.214 |
|  | H-SBP ≥145 mmHg | 232 | 3.64 (1.86–5.42) | 0.551 |

^a^*p*-value for the difference of incidence rates in the Poisson regression model.

CI, confidence interval; CV, cardiovascular; DOAC, direct oral anticoagulant; H-SBP, home systolic blood pressure; ICH, intracranial hemorrhage; Ref, reference; SEE, systemic embolic events.

**Supplementary Table 4**. Adjusted hazard ratios of events for DOAC vs warfarin by H-SBP category (analysis excludes patients with off-label DOAC doses)

| Event | Type of anticoagulant | H-SBP  <125 mmHg | | | H-SBP  ≥125 to <135 mmHg | | | H-SBP  ≥135 to <145 mmHg | | | H-SBP  ≥145 mmHg | | | *p*-value for interaction |
| --- | --- | --- | --- | --- | --- | --- | --- | --- | --- | --- | --- | --- | --- | --- |
|  |  | n | HR (95% CI) | *p*-value | n | HR (95% CI) | *p*-value | n | HR (95% CI) | *p*-value | n | HR (95% CI) | *p*-value |  |
| Net CV outcome | Warfarin^a^ | 455 |  |  | 336 |  |  | 185 |  |  | 116 |  |  | 0.876 |
|  | DOAC | 1163 | 0.98 (0.52–1.85) | 0.945 | 926 | 1.01 (0.50–2.04) | 0.975 | 509 | 1.06 (0.38–2.97) | 0.916 | 232 | 0.28 (0.09, 0.89) | 0.031 |  |
| Stroke/  SEE | Warfarin^a^ | 455 |  |  | 336 |  |  | 185 |  |  | 116 |  |  | 0.977 |
|  | DOAC | 1163 | 1.09 (0.49–2.41) | 0.840 | 926 | 1.15 (0.49–2.65) | 0.752 | 509 | 1.21 (0.29–5.01) | 0.790 | 232 | 0.38 (0.09–1.67) | 0.202 |  |
| Major bleeding | Warfarin^a^ | 455 |  |  | 336 |  |  | 185 |  |  | 116 |  |  | 0.719 |
|  | DOAC | 1163 | 1.12 (0.38–3.33) | 0.833 | 926 | 0.94 (0.27–3.27) | 0.921 | 509 | 1.31 (0.25–6.86) | 0.750 | 232 | 0.00 (0.00–0.52) | 0.029 |  |
| ICH | Warfarin^a^ | 455 |  |  | 336 |  |  | 185 |  |  | 116 |  |  | 0.779 |
|  | DOAC | 1163 | 0.65 (0.20–2.10) | 0.475 | 926 | 1.74 (0.30–10.02) | 0.538 | 509 | 5.08 (0.32–81.05) | 0.250 | 232 | -^b^ | - |  |
| All-cause death | Warfarin^a^ | 455 |  |  | 336 |  |  | 185 |  |  | 116 |  |  | 0.904 |
|  | DOAC | 1163 | 1.13 (0.72–1.76) | 0.597 | 926 | 1.05 (0.59–1.87) | 0.869 | 509 | 0.61 (0.27–1.37) | 0.233 | 232 | 0.95 (0.38–2.38) | 0.909 |  |

^a^Reference.

^b^Not calculated due to the low number of events.

CI, confidence interval; CV, cardiovascular; DOAC, direct oral anticoagulant; ICH, intracranial hemorrhage; OAC, oral anticoagulant; H-SBP, home systolic blood pressure; HR, hazard ratio; SEE, systemic embolic events.
